# Behavioral Mechanisms That Mediate Mental and Physical Health Improvements in People With Chronic Pain Who Receive a Digital Health Intervention: Prospective Cohort Pilot Study

**DOI:** 10.2196/51422

**Published:** 2023-11-17

**Authors:** Abby L Cheng, Mansi Agarwal, Melissa A Armbrecht, Joanna Abraham, Ryan P Calfee, Charles W Goss

**Affiliations:** 1 Division of Physical Medicine and Rehabilitation Department of Orthopaedic Surgery Washington University School of Medicine St Louis, MO United States; 2 Institute for Informatics, Data Science and Biostatistics Washington University School of Medicine St Louis, MO United States; 3 Department of Anesthesiology and Institute for Informatics Washington University School of Medicine St Louis, MO United States; 4 Division of Hand and Wrist Department of Orthopaedic Surgery Washington University School of Medicine St Louis, MO United States

**Keywords:** digital mental health intervention, chronic musculoskeletal pain, anxiety, depression, pain interference, physical function, behavioral activation, pain acceptance, sleep quality, mediation analysis, behavioral mechanism, chronic pain, digital health intervention, mobile phone

## Abstract

**Background:**

Preliminary evidence suggests that digital mental health intervention (Wysa for Chronic Pain) can improve mental and physical health in people with chronic musculoskeletal pain and coexisting symptoms of depression or anxiety. However, the behavioral mechanisms through which this intervention acts are not fully understood.

**Objective:**

The purpose of this study was to identify behavioral mechanisms that may mediate changes in mental and physical health associated with use of Wysa for Chronic Pain during orthopedic management of chronic musculoskeletal pain. We hypothesized that improved behavioral activation, pain acceptance, and sleep quality mediate improvements in self-reported mental and physical health.

**Methods:**

In this prospective cohort, pilot mediation analysis, adults with chronic (≥3 months) neck or back pain received the Wysa for Chronic Pain digital intervention, which uses a conversational agent and text-based access to human counselors to deliver cognitive behavioral therapy and related therapeutic content. Patient-reported outcomes and proposed mediators were collected at baseline and 1 month. The exposure of interest was participants’ engagement (ie, total interactions) with the digital intervention. Proposed mediators were assessed using the Behavioral Activation for Depression Scale–Short Form, Chronic Pain Acceptance Questionnaire, and Athens Insomnia Scale. Outcomes included Patient-Reported Outcomes Measurement Information System Anxiety, Depression, Pain Interference, and Physical Function scores. A mediation analysis was conducted using the Baron and Kenny method, adjusting for age, sex, and baseline mediators and outcome values. *P*<.20 was considered significant for this pilot study.

**Results:**

Among 30 patients (mean age 59, SD 14, years; 21 [70%] female), the mediation effect of behavioral activation on the relationship between increased intervention engagement and improved anxiety symptoms met predefined statistical significance thresholds (indirect effect –0.4, 80% CI –0.7 to –0.1; *P*=.13, 45% of the total effect). The direction of mediation effect was generally consistent with our hypothesis for all other proposed mediator or outcome relationships, as well.

**Conclusions:**

In a full-sized randomized controlled trial of patients with chronic musculoskeletal pain, behavioral activation, pain acceptance, and sleep quality may play an important role in mediating the relationship between use of a digital mental health intervention (Wysa for Chronic Pain) and improved mental and physical health.

**Trial Registration:**

ClinicalTrials.gov NCT05194722; https://clinicaltrials.gov/ct2/show/NCT05194722

## Introduction

### Background

Chronic pain commonly coexists with depression and anxiety, and these conditions are bidirectional risk factors for one another [1-5]. Fortunately, behavioral interventions such as cognitive behavioral therapy and sleep interventions can simultaneously improve symptoms related to mental health and chronic pain [6-9]. Three behavioral targets that may mediate these changes include: (1) behavioral activation, which is the process of identifying and engaging in activities that are meaningful and enjoyable, regardless of a person’s current symptom state [10-13]; (2) pain acceptance, which is “acknowledging that one has pain, giving up unproductive attempts to control pain, acting as if pain does not imply disability, and [committing] one’s efforts toward living a satisfying life despite pain” [14-19]; and (3) sleep quality, which includes a person’s pattern of falling asleep, staying asleep, total sleep duration, sleep architecture, and restorative effect of sleep [20-24]. Our preliminary work suggests that a digital mental health intervention (Wysa for Chronic Pain) which was designed to address these 3 behavioral targets can improve self-reported mental and physical health in patients who present for orthopedic management of chronic musculoskeletal pain and have coexisting symptoms of depression and anxiety [25,26]. However, mediation effects by these 3 behavioral mechanisms through which this intervention is proposed to act have not been formally tested. Before a full-sized randomized controlled trial is conducted to determine the effectiveness of this digital mental health intervention, a better understanding of the behavioral targets which it engages is needed.

### Goal of This Study

The purpose of this pilot study was to identify whether there is preliminary evidence that the behavioral mechanisms of behavioral activation, pain acceptance, and sleep quality mediate the relationship between increased engagement with a digital mental health intervention (Wysa for Chronic Pain) and improvements in mental and physical health among patients who present for orthopedic management of chronic musculoskeletal pain. We hypothesized that improved behavioral activation, pain acceptance, and sleep quality would mediate the relationship between increased engagement with the digital mental health intervention and improved self-reported anxiety, depression, pain interference, and physical function at 1 month.

## Methods

### Design

This was a single-site, single-arm, prospective cohort pilot study that was conducted at a tertiary care academic medical center in the United States. Participants were enrolled from January 2022 through May 2022. This study was prospectively registered through ClinicalTrials.gov (NCT05194722). Participants in this study also participated in a broader qualitative investigation regarding whether and how to address mental health in the orthopedic care setting [27,28].

### Ethical Considerations

Institutional review board approval (IRB #: 202110165) was obtained from he Washington University Institutional Review Board prior to participant recruitment. Participants provided written consent prior to participation, and they received remuneration for participation in the form of a US $40 gift card. To maintain participants’ privacy and confidentiality, all study data were stored in a secure electronic REDCap (Research Electronic Data Capture; Vanderbilt University) database [29,30].

### Participants

Participants were recruited from patients who presented to an orthopedic spine specialist within the institution’s orthopedic department. Eligibility criteria were assessed via medical record review and confirmation with the patient. Potential participants were contacted via telephone prior to their upcoming clinic visit with the orthopedic spine specialist or in person on the day of their visit. To be eligible, patients had to be an adult (18 years or older) who presented for a new or return evaluation of chronic (≥3 months) neck or back pain. Patients were excluded if they presented for a routine postoperative visit, endorsed active mental health crisis (eg, suicidal or homicidal ideation or psychosis), or had cognitive impairment which would interfere with engaging in this study’s surveys or intervention. The treating spine specialists included 11 physical medicine and rehabilitation physicians (physiatrists) with subspecialty training in pain medicine or sports medicine, 4 subspecialty-trained orthopedic spine surgeons, and 1 nurse practitioner who worked closely with the orthopedic spine surgery service.

### Intervention

Study participants received a 1-month subscription to Wysa for Chronic Pain, which is a multicomponent digital mental health intervention that uses an artificial intelligence–based conversational agent (ie, chatbot) and human “coaches” (counselors) with master’s degrees in psychology to deliver therapeutic content including cognitive behavioral therapy, cognitive restructuring, motivational interviewing, mindfulness training, deep breathing techniques, and sleep meditations to collectively improve users’ behavioral activation, pain acceptance, and sleep quality [31-35]. This intervention guides users through a week-by-week curriculum that uses behavioral activation and pain acceptance principles to encourage users to engage with things that bring joy, despite having pain. Features include daily check-ins, nightly sleep meditations, weekly progress reports, a progress roadmap, and the ability to unlock premium “reward” tool packs by engaging with the weekly reports. The intervention encourages users to engage with the stepwise curriculum at least 3 times weekly. For the purpose of this study, participants were encouraged to use the intervention as much as they felt was helpful. Participants also received usual orthopedic care during the course of this study, as prescribed by their orthopedic clinician. Usual orthopedic care included analgesic medication, physical therapy, and interventional spine procedures, as appropriate. No participant had spine surgery during this study’s follow-up period.

### Measures

#### Overview

Data sources included a combination of participants’ self-report, their electronic medical records, and time-stamped usage reports from the digital intervention company (Wysa Inc). At baseline, standard clinical care data were collected from the electronic medical record including participants’ age, sex, race, ethnicity, orthopedic diagnosis, and pain medications, and participants also self-reported their pain duration, general smartphone use patterns, and current use of mental health treatment (eg, medications, psychotherapy, and behavioral techniques). Proposed behavioral mechanisms of change and clinical mental and physical health outcomes of interest (measured by Patient-Reported Outcomes Measurement Information System [PROMIS]) were also collected via electronic survey at baseline and 1-month follow-up, and participants reported any changes in mental health management at 1-month follow-up. Baseline PROMIS measures were obtained from the electronic medical record because they were collected as part of usual orthopedic care. Follow-up PROMIS measures were collected 1 month later via email invitation to an electronic survey. Further, 1 month was selected as the follow-up time point of interest because patients who present for orthopedic care of chronic musculoskeletal pain generally expect to start noticing clinical improvements related to conservative management and recommended behavior changes within 1 month.

#### Exposure—Intervention Engagement

Quantitative usage of the digital mental health intervention (Wysa for Chronic Pain) was determined by time-stamped data for each of participants’ interactions with the intervention’s conversational agent (ie, chatbot), other digital tools, or human coach. The exposure of interest was defined as the participants’ total number of interactions with the intervention during the 1-month study period, including all digital and human coach interactions. These data were provided to this study’s team via random user identifiers which ensured that only this study’s team could link participants’ usage data with their identities and other study data. Personally identifiable information was not shared between Wysa Inc and this study’s team.

#### Proposed Mediators—Behavioral Targets

The proposed behavioral mechanisms of action were operationalized by 3 self-reported measures. Behavioral activation was quantified with the Behavioral Activation for Depression Scale–Short Form (BADS-SF), which is scored from 0 to 54, with higher scores being favorable and representing more activation [36]. Pain acceptance was quantified with the 8-item Chronic Pain Acceptance Questionnaire, which is scored from 0 to 48, with higher scores being favorable and representing more pain acceptance [37]. Sleep quality was quantified with the Athens Insomnia Scale, which inquires about participants’ ability to fall asleep, stay asleep, total sleep duration, and restorative nature of sleep. It is scored from 0 to 24, with lower scores being favorable [38,39].

#### Outcomes—Mental and Physical Health

The clinical outcomes of interest were changes in self-reported mental and physical health, as measured by the adult PROMIS computer adaptive test Anxiety (version 1.0), Depression (version 1.0), Pain Interference (version 1.1), and Physical Function (version 2.0) measures [40-43]. Scoring for each PROMIS measure is normalized to the general US population with a mean of 50 and SD of 10. Higher scores represent more of the domain being measured. Therefore, lower scores are favorable for PROMIS Anxiety, Depression, and Pain Interference, and higher scores are favorable for PROMIS Physical Function [44]. For reference, clinically meaningful PROMIS score changes in patients with conservatively managed chronic musculoskeletal pain have previously been defined as 3.0 points for Anxiety, 3.2 points for Depression, 2.0 points for Pain Interference, and 2.2 points for Physical Function [45-47].

### Statistical Analysis

The primary study outcome was whether preliminary evidence exists for the hypothesized mediation relationships of the proposed mediators on the exposure-outcome relationship between intervention engagement and self-reported (mental and physical) health. Using the approach from Baron and Kenny [48], the following criteria were used to evaluate our mediation analyses: (1) the exposure is significantly associated with the mediator variable, (2) the exposure is significantly associated with the outcome (total effect), (3) the mediator is significantly associated with the outcome when the exposure is also included in the model, and (4) the effect of the exposure on the outcome is attenuated when the mediator is also included in the model. The exposure (ie, intervention engagement) was natural log transformed to reduce skewness in the data, with +1 added to account for zeroes. The analyses were adjusted for baseline values for the proposed mediators and the outcomes, as well as for age and sex since these are potential confounders of the relationship between the exposures and outcomes. In addition to the regression coefficients, we also report the percentage of the total effect accounted for by the exposure and mediator to facilitate interpretation. Since this is a pilot trial, we considered *P*<.20 as a “significant” effect and reported 80% CIs to align with this threshold [49,50]. The sample size for this pilot study was determined by the availability of resources. There were no missing data. Statistical analyses were performed using SAS (version 9.4; SAS Institute Inc).

## Results

**Figure 1:**
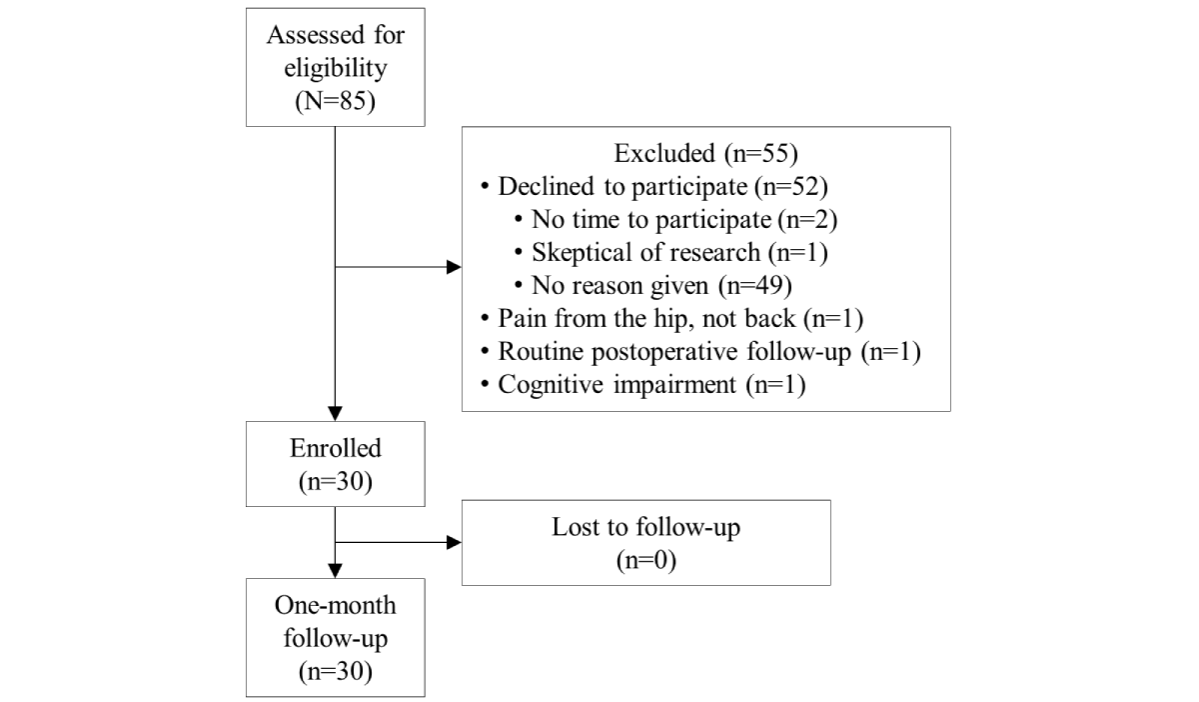
Inclusion flowsheet of patients who sought orthopedic care for chronic neck or back pain between January 2022 and May 2022.

In total, 30 of 85 (35%) eligible patients were enrolled, and all 30/30 (100%) completed follow-up measures (Figure 1). The cohort had a mean age of 59 (SD 14) years, and 21/30 (70%) were female (Table 1). During the 1-month study period, 28/30 (93%) engaged with the digital mental health intervention at least once, and 9/30 (30%) engaged at least once with the human coach of the digital intervention (Table 2). The majority of participants (24/30, 80%) did not make any other mental health management changes during the follow-up period. On average for the entire cohort, the directionality of change for all proposed mediators and clinical outcomes was consistent with improvement (Table 3). Among the proposed mediators, the greatest change was in behavioral activation (BADS-SF mean change 4.5 points, 80% CI 2.9 to 6.1). Among the clinical outcomes, clinically meaningful changes were observed for PROMIS Anxiety (–3.2, –4.3 to –2.0 points) and PROMIS Pain Interference (–3.5, –4.8 to –2.2 points).

**Figure 2:**
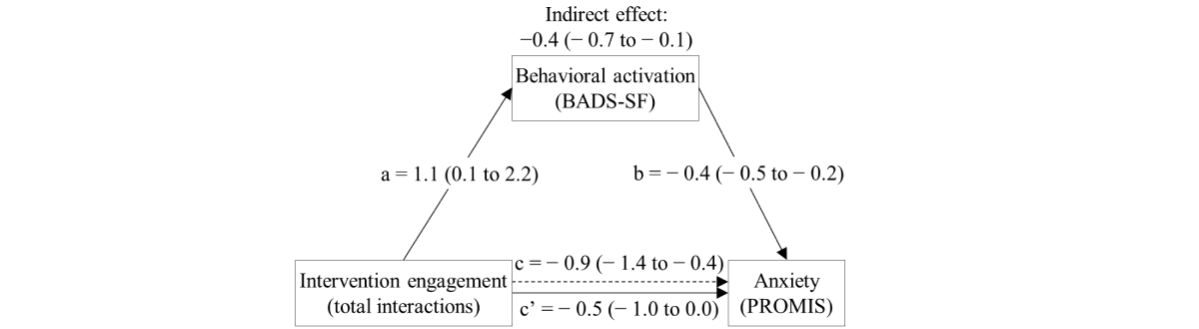
Mediation effect of behavioral activation on the relationship between intervention engagement and anxiety symptoms over a 1-month time interval among 30 patients who presented to an orthopedic clinic for management of chronic neck or back pain between January 2022 and May 2022. “a” represents the slope estimate between the exposure (intervention engagement) and proposed mediator (behavioral activation). “b” represents the slope estimate of the proposed mediator (behavioral activation) in the full model that includes the exposure (intervention engagement) and all covariates. Parentheses depict 80% CIs. Covariates include age, sex, and baseline BADS-SF and PROMIS Anxiety scores. c’: total direct effect; c: total effect; BADS-SF: Behavioral Activation for Depression Scale–Short Form; PROMIS: Patient-Reported Outcomes Measurement Information System.

The mediation effect of behavioral activation on the relationship between increased intervention engagement and improved anxiety symptoms met our statistical threshold for significance (indirect effect –0.4, 80% CI –0.7 to –0.1; *P*=.13; Figure 2). Behavioral activation mediated 45% of this relationship. Although not statistically significant, the mediation analyses also revealed that the direction of mediation effect was generally consistent with our hypothesis for all other mediator or outcome relationships (Table 4). Specifically, improvements in clinical outcomes (ie, PROMIS Anxiety, Depression, Pain Interference, and Physical Function) were associated with increased engagement with the digital mental health intervention (Wysa for Chronic Pain) and were partially mediated by improvement in our proposed mediators (ie, behavioral activation [BADS-SF], pain acceptance [Chronic Pain Acceptance Questionnaire], and sleep quality [Athens Insomnia Scale]), with the exception that pain acceptance did not demonstrate any mediation effect on the relationship between intervention engagement and pain interference or physical function.

**Table 1:** Baseline characteristics of 30 patients who presented to an orthopedic clinic for management of chronic neck or back pain between January 2022 and May 2022.

Characteristics			n (%)
**Age (years), mean (SD; range)**			59 (14; 30 to 78)
**Sex**			
	Female	21 (70)	
	Male	9 (30)	
**Race**			
	White	18 (60)	
	Black	10 (33)	
	Asian	2 (7)	
**Ethnicity**			
	Hispanic	1 (3)	
	Not Hispanic	29 (97)	
**Smartphone use**			
	Independent downloading and using apps	17 (57)	
	Need help with downloading and using apps	6 (20)	
	Smartphone user but not for apps	5 (17)	
	Never use a smartphone	2 (7)	
**Pain duration (years), mean (SD; range)**			8.6 (10.7; 0.5 to 40.0)
**Orthopedic diagnosis**			
	Spinal stenosis	11 (37)	
	Radiculopathy	11 (37)	
	Facet arthropathy	3 (10)	
	Spondylosis	9 (30)	
	Scoliosis	2 (7)	
	Myofascial pain	3 (10)	
**Current treatments for mental health**			
	Medication	9 (30)	
	Psychiatrist, psychologist, or counselor	5 (17)	
	Support group	1 (3)	
	Mental health app	0 (0)	
	Self-management (meditation and mindfulness)	14 (47)	
	Family support	2 (7)	
	None	10 (33)	
	Prefer not to answer	1 (3)	
**Medications taken for pain**			
	Opioid	4 (18)	
	Tramadol	3 (14)	
	Neuropathic (eg, gabapentin)	10 (46)	
	Muscle relaxant (eg, cyclobenzaprine)	9 (41)	
	Oral corticosteroid taper	4 (18)	
	Nonsteroidal anti-inflammatory	13 (59)	
	Other (eg, topical analgesic)	2 (9)	

**Table 2:** Engagement with mental health resources during the 1-month study period among 30 patients who presented to an orthopedic clinic for management of chronic neck or back pain between January 2022 and May 2022.

Characteristics			n (%)
**Engagement with digital mental health intervention**			
	Completed onboarding	28 (93)	
	Engaged with human coach at least once	9 (30)	
	Total interactions^a,b^, median (IQR; range)	19 (7 to 64; 1 to 382)	
	Total interactions with chatbot^a^, median (IQR; range)	14 (5 to 40; 1 to 205)	
	Total interactions with coach^a^, median (IQR; range)	0 (0 to 1; 0 to 7)	
**Other change in mental health management**			
	Added a medication	1 (3)	
	Started psychotherapy	6 (20)	
	No change	24 (80)	

^a^n=28 for intervention interactions because 2 participants did not complete onboarding.

^b^Total interactions (ie, digital tools plus human coach sessions) with the digital mental health intervention is the exposure of interest for the mediation analyses.

**Table 3:** Longitudinal changes in proposed mediators and clinical outcomes during the 1-month study period among 30 patients who presented to an orthopedic clinic for management of chronic neck or back pain between January 2022 and May 2022.

Variable		Baseline, mean (SD; range)	One month, mean (SD; range)	Mean change (80% CI)^a^	*P* value
**Proposed mediators—Behavioral targets**					
	BADS-SF^b^ total score	26.7 (8.3; 12.0 to 52.0)	31.2 (8.9; 16.0 to 53.0)	4.5 (2.9 to 6.1)	<.001
	CPAQ-8^c^ total score	21.2 (7.0; 5.0 to 33.0)	23.7 (6.6; 12.0 to 36.0)	2.5 (1.2 to 3.7)	.014
	AIS^d^ total score	11.4 (6.3; 3.0 to 24.0)	9.5 (5.1; 3.0 to 18.0)	–2.0 (–3.1 to –0.8)	.03
**Outcomes—PROMIS^e^ Scores**					
	Anxiety	59.1 (8.9; 38.3 to 73.3)	56.0 (7.4; 35.9 to 67.2)	–3.2 (–4.3 to –2.0)	<.001
	Depression	52.9 (8.7; 34.2 to 69.5)	51.6 (8.0; 34.2 to 64.0)	–1.3 (–2.9 to 0.3)	.31
	Pain Interference	64.2 (7.2; 50.1 to 80.0)	60.7 (6.9; 50.1 to 83.8)	–3.5 (–4.8 to –2.2)	.002
	Physical Function	38.6 (7.2; 26.9 to 58.9)	39.9 (5.6; 30.0 to 51.2)	1.4 (–0.2 to 2.9)	.26

^a^Mean changes were calculated using paired *t* tests.

^b^BADS-SF: Behavioral Activation for Depression Scale–Short Form.

^c^CPAQ-8: Chronic Pain Acceptance Questionnaire.

^d^AIS: Athens Insomnia Scale.

^e^PROMIS: Patient-Reported Outcomes Measurement Information System.

**Table 4:** Total and constituent effects of digital mental health intervention engagement (exposure) on PROMIS score changes (outcomes) over a 1-month time interval among 30 patients who presented to an orthopedic clinic for management of chronic neck or back pain between January 2022 and May 2022.^a,b^

Outcome			Total effect				Direct effects^c^				Indirect effects^d^		
PROMIS^e^ domain			Estimate (80% CI)		*P* value		Estimate (80% CI)		*P* value		Estimate (80% CI)		*P* value
**Proposed mediator: behavioral activation (BADS-SF^f^)**													
	Anxiety	–0.9 (–1.4 to –0.4)		.03^g^		–0.5 (–1.0 to 0.0)		.16^g^		–0.4 (–0.7 to –0.1)		.13^g^	
	Depression	–0.7 (–1.5 to 0.2)		.32		–0.1 (–0.9 to 0.7)		.90		–0.6 (–1.0 to –0.1)		.12^g^	
	Pain interference	0.1 (–0.6 to 0.9)		.83		0.4 (–0.3 to 1.1)		.47		–0.3 (–0.6 to 0.1)		.35	
	Physical function	0.5 (–0.3 to 1.2)		.41		0.3 (–0.5 to 1.0)		.63		0.2 (–0.1 to 0.5)		.32	
**Proposed mediator: pain acceptance (CPAQ-8^h^)**													
	Anxiety	–0.8 (–1.4 to –0.2)		.08^g^		–0.7 (–1.3 to –0.1)		.11^g^		–0.1 (–0.2 to 0.1)		.49	
	Depression	–0.5 (–1.4 to 0.4)		.50		–0.4 (–1.3 to 0.5)		.59		–0.1 (–0.3 to 0.1)		.53	
	Pain interference	0.0 (–0.7 to 0.7)		.99		0.0 (–0.7 to 0.7)		.97		0.0 (–0.1 to 0.2)		.94	
	Physical function	0.2 (–0.4 to 0.8)		.71		0.2 (–0.4 to 0.8)		.71		0.0 (0.0 to 0.0)		.95	
**Proposed mediator: sleep quality (AIS^i^)**													
	Anxiety	–0.8 (–1.3 to –0.3)		.06^g^		–0.6 (–1.1 to –0.1)		.11^g^		–0.1 (–0.4 to 0.1)		.36	
	Depression	–0.5 (–1.3 to 0.4)		.49		–0.2 (–1.1 to 0.6)		.73		–0.2 (–0.5 to 0.1)		.32	
	Pain interference	–0.2 (–0.9 to 0.5)		.75		0.0 (–0.6 to 0.6)		.99		–0.2 (–0.6 to 0.2)		.59	
	Physical function	0.4 (–0.3 to 1.1)		.41		0.2 (–0.4 to 0.9)		.64		0.2 (–0.1 to 0.5)		.38	

^a^For a mediation effect to be considered statistically significant, both the total and indirect effects for a given mediator or outcome pair must meet statistical significance.

^b^Models are adjusted for age, sex, and baseline scores for the relevant proposed mediator (Behavioral Activation for Depression Scale–Short Form [BADS-SF], Chronic Pain Acceptance Questionnaire [CPAQ-8], or Athens Insomnia Scale [AIS]) and Patient-Reported Outcomes Measurement Information System [PROMIS] domain (Anxiety, Depression, Pain Interference, or Physical Function).

^c^Direct effects represent the effect attributed to the exposure (ie, intervention engagement).

^d^Indirect effects represent the effect attributed to the proposed mediator (ie, BADS-SF, CPAQ-8, or AIS).

^e^PROMIS: Patient-Reported Outcomes Measurement Information System.

^f^BADS-SF: Behavioral Activation for Depression Scale–Short Form.

^g^these *P* values indicate *P*<.20.

^h^CPAQ-8: Chronic Pain Acceptance Questionnaire.

^i^AIS: Athens Insomnia Scale.

## Discussion

### Principal Results

The purpose of this pilot trial was to identify whether there is preliminary evidence that the mechanisms of behavioral activation, pain acceptance, and sleep quality mediate the relationship between increased engagement with a digital mental health intervention (Wysa for Chronic Pain) and improvements in mental and physical health among patients who present for orthopedic management of chronic musculoskeletal pain. We found preliminary evidence that behavioral activation partially mediates the relationship between increased intervention engagement and improved anxiety symptoms (indirect effect –0.4, 80% CI –0.7 to –0.1; *P*=.13, 45% of the total effect). Our hypothesis was also somewhat supported in that the directionality of most of the mediation analyses suggested that improvements in self-reported mental and physical health outcomes were associated with increased intervention engagement and were partially mediated by improvement in our proposed mediators of behavioral activation, pain acceptance, and sleep quality. Nevertheless, the majority of our analyses did not meet our a priori specified statistical significance of *P*<.20.

### Interpretations

Given the pilot nature and small sample size of this study cohort, our findings suggest that a larger trial is needed to confirm these preliminary results. Worth particular mention, however, is that mediation analyses for the outcome of depression were limited due to the modest relationship (ie, small total effect sizes) observed between intervention engagement and PROMIS Depression score changes. We suspect this is, in part, related to participants’ relatively minimal reported depression symptoms at baseline, which likely also contributed to minimal improvement in depression symptom severity at 1-month follow-up. The modest changes in depression symptom severity may also be related to this study’s short follow-up period of 1 month. Our previous pilot work related to this digital mental health intervention suggested that depression symptoms continued to improve between 1- and 2-month follow-ups [25].

### Comparison With Prior Work

This study complements prior studies performed by Vranceanu’s team [51,52] which showed that pain catastrophizing, kinesiophobia, mindfulness, and pain resilience are mediators of mental and physical health in people with chronic pain. The behavioral target of pain acceptance that we investigated is related to these other mechanisms because pain acceptance addresses: (1) pain catastrophizing, which is an exaggerated cognitive or emotional response to actual or anticipated pain [51-53], and (2) kinesiophobia, which is the avoidance of activities or movements due to excessive anxiety that it will cause pain or injury [54]. Our study is different from Vranceanu’s because, in contrast to her study: (1) all participants in our study were actively seeking treatment for chronic musculoskeletal pain, and (2) our intervention was less intensive, yet intervention use was still preliminarily associated with changes in our hypothesized behavioral mechanisms and clinical outcomes.

### Limitations

This study was limited by a small sample size, lack of comparison intervention arm, short follow-up duration of 1 month, and minimal reported depression symptoms at baseline. The small sample size contributed to large CIs, and this study evaluated multiple comparisons and was not powered to detect prespecified mediation effects. Therefore, although *P* values were reported to facilitate rapid interpretation of the data, they should not be interpreted as definitive evidence of statistical significance. The lack of comparison arm, short follow-up duration, and minimal reported depression symptoms at baseline may also have affected the magnitude of clinical improvements observed during this study’s period. Nevertheless, clinical improvements were made in some domains at this time point, and this pilot study generated the necessary preliminary evidence regarding mediation effects to appropriately plan for a fully powered randomized controlled trial. Another limitation that should be addressed in a rigorous clinical trial is that, in this study, mediation and outcome measures were only collected at 2 time points, so it was not possible to assess whether improvements in proposed behavioral mediators preceded improvements in clinical outcomes.

### Conclusions

We found preliminary evidence that behavioral activation partially mediates the relationship between increased engagement with a digital mental health intervention (Wysa for Chronic Pain) and improved self-reported anxiety symptoms. There is also less strong but directionally consistent evidence suggestive of mediation by pain acceptance and sleep quality on the relationship between increased intervention engagement and improvements in mental and physical health, with the exception of lack of evidence for a mediating effect of pain acceptance on pain interference or physical function. In a full-sized randomized controlled trial of patients with chronic musculoskeletal pain, it will be worthwhile to consider behavioral activation, pain acceptance, and sleep quality as hypothesized mediators of the relationship between engagement with a digital mental health intervention (Wysa for Chronic Pain) and improved self-reported mental and physical health.

## Abbreviations

BADS-SF: Behavioral Activation for Depression Scale–Short Form
PROMIS: Patient-Reported Outcomes Measurement Information System
REDCap: Research Electronic Data Capture

## Data Availability

The data set used and analyzed during this study are available from the corresponding author on reasonable request.
